# Assessment of the content validity of the Oswestry Disability Index (ODI) in patients with Degenerative Disc Disease (DDD): a qualitative study

**DOI:** 10.1186/s12891-025-09324-1

**Published:** 2026-01-08

**Authors:** John H. Powers, Rachel Ballinger, Andrea De Palma, Marie de la Cruz, Kellee Howard

**Affiliations:** 1 George Washington University School of Medicine, Washington, DC USA; 2 ICON Insights, Evidence and Value, Reading, UK; 3 Formerly ICON Insights, Evidence and Value, Milan, Italy; 4 Present Address: Erasmus School of Health Policy and Management, Erasmus University Rotterdam, Rotterdam, The Netherlands; 5 ICON Insights, Evidence and Value, Raleigh, NC USA; 6 Formerly ICON Insights, Evidence and Value, Quebec City, Canada; 7 Present Address: IQVIA, Montreal, QC Canada

**Keywords:** Degenerative disc disease, Low back pain, Pain measurement, Oswestry disability index, Content validity, Electronic version

## Abstract

**Background:**

Degenerative disc disease (DDD) is a common disorder that can lead to chronic lower back pain (CLBP). The Oswestry Disability Index (ODI) is a well-established instrument for diseases of the lumbar spine and lower back pain (LBP), covering pain intensity and pain-related disability. This study evaluated the content validity of the modified electronic ODI among patients with DDD and CLBP in the United States (US).

**Method:**

Cognitive interviews were conducted with 12 participants from the US diagnosed with DDD and CLBP using a semi-structured interview guide to assess the comprehension and relevance of the ODI. Concept saturation was achieved by the 12th interview.

**Results:**

Twelve participants (mean age 40 years old, standard deviation 11 years) including 11 females provided interpretations of the instructions and for each section that were aligned with the intended meanings (11–12/12, ≥ 92%). Sections were relevant to participants’ experience (11–12/12, ≥ 92%), with the sex life section being less relevant (9/12, 75%). Response options were easy to understand. Participants’ interpretations aligned with response options’ intended meanings in 7/10 sections, with less alignment in the lifting, social life, sex life sections due to perceived similarities in ≥ 2 response options. Participants used different recall periods across sections, which was often related to the absence of experiencing the activity at the time of the interview. Most (7/9, 78%) preferred the use of a one-week recall period.

**Conclusions:**

This study confirms the content validity of the ODI in patients with DDD. The ODI with a seven-day recall period would be more appropriate for use in this population to evaluate patient outcomes in a clinical trial.

## Background

Degenerative disc disease (DDD) is a commonly diagnosed disorder that can lead to chronic lower back pain (CLBP), radicular symptoms, and nerve compression [1, 2], and may be exacerbated by position and movement [3]. DDD occurs in an estimated 40% of individuals under 30 years of age and at least 90% of individuals older than 50 years of age. DDD is influenced by vascular supply, genetics, biomechanics, infection, and trauma [1, 3]. Lumbar disc disease carries enormous morbidity, leading to disability, socioeconomic impacts, and a poor quality of life [4]. Patient history and report of symptoms play a key role in the diagnosis of DDD as the various diagnostic modalities (e.g. MRIs) when used alone are not definitive for the diagnosis of pain [5, 6] .

In a clinical trial setting, it is important to demonstrate the therapeutic benefit of a treatment on direct outcomes relevant to patients. The effect of a treatment is often measured using reliable patient-reported outcome measures (PROMs). These can be either condition-specific or generic measures and they are used to assess symptoms and patient functioning captured directly from patients [7]. Generic pain measures can be multidimensional instruments such as the Brief Pain Inventory and the Multidimensional Pain Inventory as well as a visual analogue scales (VAS) or a numeric rating scales (NRS). Their advantage is their well-documented evidence on validation and ease of administration [8]. However, these measures are not developed for DDD and do not evaluate the complete experience of patients with DDD [9]. With patients with DDD, both pain intensity and pain related disability were identified as patient-centered concepts of interest [10–20]. Within clinical trials, improvements in both must be measured in order to provide evidence of a clinically meaningful treatment benefit.

**Fig. 1:**
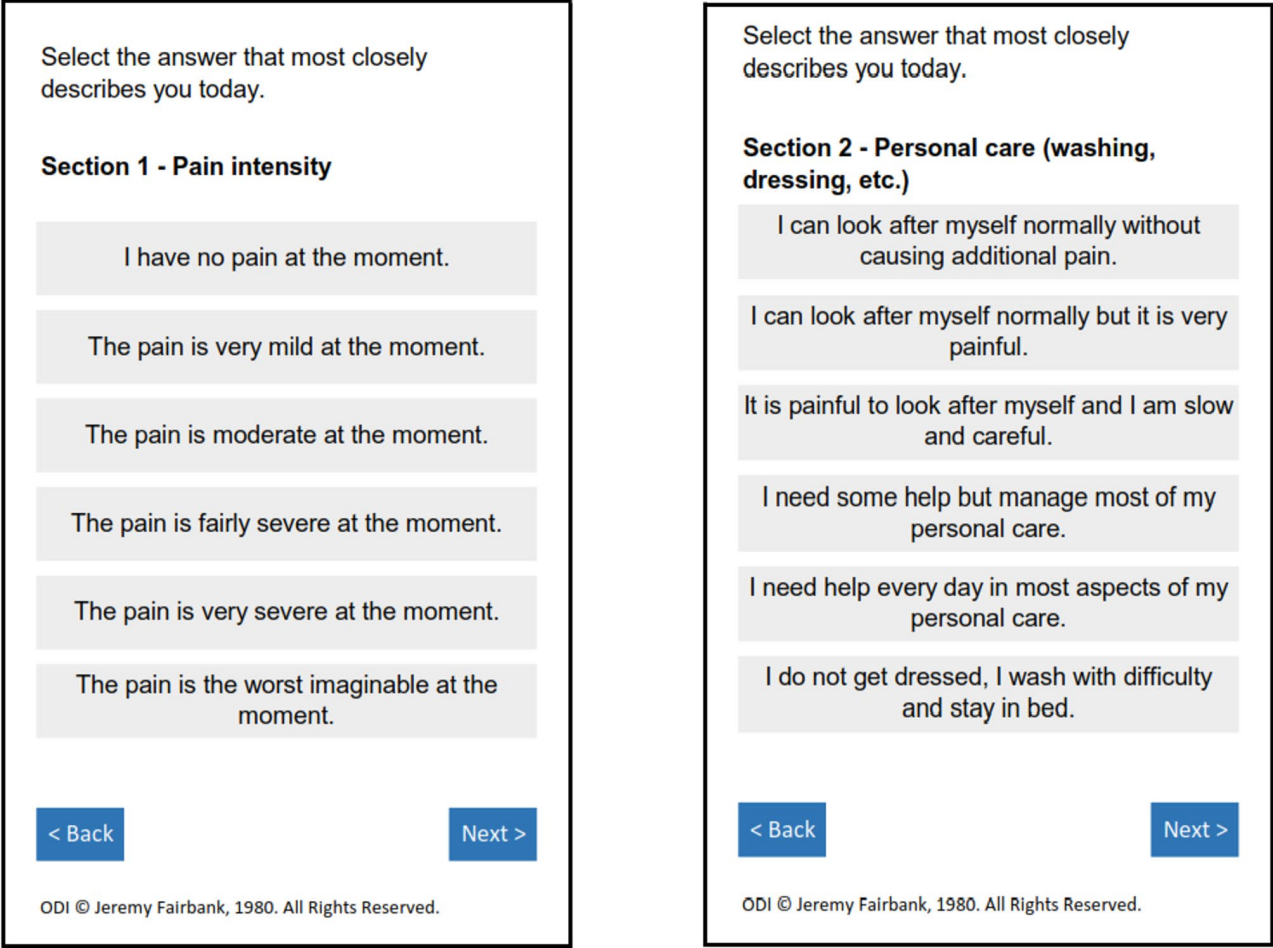
Example screenshots from ODI v2.1. Sample copy, do not use without permission. Original questionnaire reference: Fairbank JC, Couper J, Davies JB, O’Brien JP. The Oswestry Low Back Pain Disability Questionnaire. Physiotherapy. 1980;66(8):271–273. ODI contact information and permission to use: Mapi Research Trust, Lyon, France, https://eprovide.mapi-research-trust-org

The Oswestry Disability Index (ODI) is a well validated PROM that covers pain intensity and pain-related disability [21]. It is one of the most established instruments for diseases of the lumbar spine [9]. The ten questions in the ODI were originally developed with physician and patient input from 1976 to 1980 [22]. Patients who were referred to a specialty clinic for CLBP were interviewed to understand concepts of interest for these patients. A team of expert physicians comprising spine surgeons, occupational therapists and physical therapists constructed the ODI questions based on this input [22, 23]. The questions were reviewed, tested and iterated using patient input [22]. The final ODI is comprised of 10 sections (single items about pain intensity, personal care, lifting, walking, sitting, standing, sleeping, sex life, social life, travelling). Each section has six different descriptive response options (Fig. 1). Respondents are instructed to answer each section using a ‘today’ recall period. The ODI’s psychometric properties have been well established, demonstrating good construct validity (validation of the Pain Disability Index, the Low Back Outcome Score, the Manniche Scale, the Aberdeen Score, the Curtin Scale, was performed with the ODI), acceptable internal consistency (Cronbach’s alpha coefficient 0.71–0.99), and high test-retest reliability (correlation coefficient *r* = 0.83–0.99, *p* < 0.001) and responsiveness (minimum clinically important difference [MCID] ranges between 10.5 and 15 points) [22, 24, 25]. In addition, the instrument has been translated for use in a variety of countries including France, Germany, Italy, Korea, China, Russia, Argentina, Mexico, Sweden, India, and Vietnam [26].

The ODI is one of the reference standard PROMs in patients with CLBP and has been used to validate multiple other CLBP PROM’s with relevant a priori concepts [27–35]. The ODI has also been recommended for use by several groups and initiatives in assessing treatment effects in patients with CLBP [33–36]. In particular, through the guidelines of the Low Back Pain Initiative, the World Health Organization (WHO) recommended the use of ODI as the PROM for standardized evaluations [36]. The International Consortium for Health Outcomes Measurement (ICHOM) (reference v2.0.3 Aug. 2017) Low Back Pain group also recommended the ODI v.2.1a as the core standard PROM for patient-reported pain-related disability. The Initiative on Methods, Measurement and Pain Assessment in Clinical Trials (IMMPACT) guidelines included the ODI as a validated disease-specific PROM for evaluating physical function in the population with CLBP [37, 38]. An expert panel initiated by Deyo and colleagues recommended the ODI in the proposed set of core instruments for functional status (i.e. pain-related disability) assessment [39]. Several reviews have confirmed its continuing prevalence in assessing treatment outcomes for low back pain [40–42] .

Since its development, the ODI has undergone several modifications following language adaptations to facilitate translation [43], with the most recent version being the ODI v2.1b. Despite its popularity, a recent systematic review of the most commonly used PROMs measuring physical functioning in patients with LBP concluded that qualitative research is urgently needed to assess ODI’s relevance, comprehensiveness, and comprehensibility for measuring physical functioning in this patient population [44]. In addition, scientific literature identifies four threats to content validity for PROMs, specifically (1) conceptual misalignment between a PROM and the desired claim; (2) missing direct patient input from the target population for which a claim is intended; (3) no evidence that a PROM contains item content that is most relevant and important; and (4) lack of documentation to support changes made to a PROM [45]. The aim of this study was to provide evidence to address each of these issues and contribute well-documented qualitative research, which can mediate threats to PROMs content validity [33–35]. In particular, cognitive interviews can illustrate participants’ interpretations of questions, the way they formulate those interpretations and highlight mismatches with the research team’s intent [46]. Furthermore, qualitative cognitive reviews are frequently undertaken to evaluate the content validity of electronic versions of previously validated PROMs [47].

The objective of this study was to gain perspective on content validity of the ODI from a sample of patients with DDD about the electronic ODI v2.1.b (US English). In particular, the study assessed the comprehension, interpretation and appropriateness of items, response categories and recall period of the modified Oswestry Disability Index (mODI, version 2.1b) among US patients diagnosed with DDD.

Using established methodologies to evaluate and document content validity [45] the study aimed to ensure that the electronic ODI v2.1.b (US English) instrument is appropriate and fit-for-purpose in measuring pain intensity and pain-related disability in a future clinical trial setting.

## Methods

Cross-sectional cognitive interviews based in content analysis were conducted with patients who reported diagnosis of DDD to assess the content validity of the modified electronic ODI based on well-established guidelines [45].

### Recruitment strategy

Patients between 18 and 55 years with CLBP and DDD diagnosis were recruited from a specialist clinical site and through a specialist recruitment vendor firm (Global Perspectives). Site staff disseminated an advertisement flyer within their specialist back pain clinic. Interested patients could make contact with the study team, via the contact details on the flyer, to request further information or share their interest in participating. In addition, the specialist recruitment vendor firm identified participants through their patient proprietary databases, physician referrals, internet and social media advertising using IRB-approved recruitment messages. Potential participants were sent a Study Information Sheet which included the eligibility criteria and consent process. Eligibility criteria included the following: aged between 18 and 55 years and a diagnosis of DDD with current experience of lower back pain for longer than 6 months that impacted their everyday abilities. Pain and impact duration reflected eligibility criterion in a planned clinical trial [48]. Participants were also asked to be able to read English, use email and be at a computer during the interview to be able to review the study documents. There were no additional exclusion criteria. Prior to the start of the interviews, participants were asked to confirm that they read the Study Information Sheet and that they met the eligibility criteria listed in the Study Information Sheet. In addition, participants were asked for their consent to audio-record the interviews.

### Research procedure

A semi-structured interview guide was developed by the study team consisting of cognitive debriefing questions to evaluate the comprehension and relevance of the complete instrument. The study protocol and materials were reviewed and approved by Salus IRB (Independent Review Board) in the US (protocol number 6005-0002, approved 06 October 2021 and amendment approved 28 October 2021). Consistent with best practices for evaluating content validity of existing instruments [45], the interview guide included questions that elicited feedback on participant understanding of the instructions, items, response options, and recall period; ease of completion; and relevance. Furthermore, the comprehensiveness of the instrument was assessed by asking participants if there were any aspects of their CLBP that were not addressed in the questionnaire. In addition, the interview guide included socio-demographic questions as well as questions of participants’ prior ODI completion experience.

### Data collection

All interviews were conducted via telephone/video conference between October and November 2021 by an experienced female interviewer with a Master of Science degree and 15 years of interview experience. Prior to interviews, the interviewer was trained on the study protocol and the interview guide. The interviewer did not have a priori knowledge or conceptions that could bias interview conduction.

Potential participants were provided with a study information sheet to help them decide if they wished to take part in the 60-minute, cognitive interviews. Eligible individuals were scheduled for remote telephone/video conferencing interviews. Participants were provided with the purpose of the study by the interviewer and were given the opportunity to ask any questions. Participants were then asked to provide their verbal consent at the start of the interview, which was captured on the audio recording. At the start of the interview participants were asked socio-demographic questions. Participants were then provided the password to open the ODI file, which comprised screenshots of the electronic version of the ODI v2.1b. Participants were asked to read through the questionnaire and provide their answers verbally to each of the sections, before being asked cognitive questions about the ODI. Upon completion of the interview, participants were reimbursed for their time and participation.

### Data analysis

An experienced interviewer used external audio devices (Olympus Digital Voice Recorder WS-852; made in Vietnam) to record the interviews. An additional study team member took contemporaneous notes during the interview. The audio recordings were stored on secure servers. The audio-recordings were transcribed (single transcription) verbatim then de-identified by the study team for analysis. An Excel file was then developed to summarize findings related to the comprehension, interpretation and appropriateness of the ODI’s items, response categories and recall period, and any other themes arising from the data. Quality control checks of the transcripts and data entry was conducted by three researchers (MDLC, RB, ADP). This entailed reviewing the transcripts and the Excel file against the original audio recordings to ensure they accurately reflected what was reported by the participants.

Analysis of the qualitative data was conducted on an item-by-item basis as outlined by Willis [49]. The transcripts and notes were reviewed and evaluated to help inform probing in subsequent interviews, identify any issues with the ODI, assess concept saturation (the point at which no new information is elicited and deemed missing during the interviews) [45], and inform the decision to undertake additional interviews. For assessment of instruction and question interpretation, participants were asked to summarize the items in their own words. Their responses were then compared to the intended meaning of the item and considered as supported or unsupported.

All participants’ socio-demographic information was summarized using descriptive statistics (e.g., means, standard deviations and frequencies).

## Results

### Sample characteristics

A total of 12 participants from the US meeting the eligibility criteria took part in the study. The participants had a mean age of 40 years (SD = 11) and were mainly female (*n* = 11/12, 92%), White/Caucasian (*n* = 10/12, 83%), married or engaged (*n* = 9/12, 75%). Half of participants had received at least some college education (*n* = 6/12, 50%). The participants had various working statuses, including being employed (*n* = 5/12, 42%), disabled, temporarily or permanently unable to work due to their condition (*n* = 5/12, 42%), or looking after their home or family (*n* = 2/12, 17%).

The mean years since diagnosis of DDD was 6 years (SD = 5), while the average number of years since initial symptoms was 12 years (SD = 8). Most participants reported never completing the ODI before (*n* = 9/12, 75%). The mean ODI score of the sample was 53% indicating that the majority of the participants have severe disability or crippling back pain (*n* = 10, 83%). A summary of the demographic characteristics is provided in Table 1.

**Table 1:** Sample Socio-Demographic information

Characteristic	Total Sample (*N* = 12) *N* (%)
Gender	
Male	1 (8%)
Female	11 (92%)
Age (years)	
Mean (SD)	40 (11)
Range	26–53
Race	
White/Caucasian	10 (83%)
African/American	2 (17%)
Highest Level of Education Completed	
Some High School	1 (8%)
High School Diploma/GED	5 (42%)
Some College	1 (8%)
Associate’s Degree	1 (8%)
Bachelor’s Degree	3 (25%)
Master’s Degree	1 (8%)
Marital Status	
Married/Engaged	9 (75%)
Single	3 (25%)
Working Status	
Full-time job	1 (8%)
Part-time job	3 (25%)
Disabled	1 (8%)
Permanently unable to work due to sickness or injury	2 (17%)
Temporarily unable to work due to sickness or injury	2 (17%)
Self-employed	1 (8%)
Looking after home or family	2 (17%)
When diagnosed with DDD (N years ago)^a^	
Mean (SD)	6 (5)
Range	1–18
When first noticing symptoms of back pain (years)^b^	
Mean (SD)	12 (8)
Range	1–25
ODI Score	
Mean (SD)	53 (13)
Range	28–68
Completed ODI before	
Yes	3 (25%)
No	9 (75%)

*SD* Standard Deviation, *GED* General Equivalency Diploma, *ODI* Oswestry Disability Index

^a^Responses from 11 participants. This question was probed from the fourth interview onwards. Data was indicated for 2 participants at screening stage

^b^Responses from 9 participants. This question was probed from the fourth interview onwards

### ODI content validity

#### ODI instructions

Participants were asked to explain the purpose of the questionnaire. Most participants (11/12, 92%) reported an accurate understanding of the intent of the ODI. Moreover, all participants (12/12, 100%) reported no difficulty in understanding the instructions and the majority (9/12, 75%) provided clear and supportive interpretations of the instructions, for example:

Asking me to give detailed information on how my back troubles affect my everyday life.

A summary of the findings per ODI section is provided in Table 2.

**Table 2:** Summary of results across all sections

Section	Question Interpretation (*N* = 12)	Response Options Understanding (*N* = 12)	Response Options Interpretation (*N* = 10)^a^	Any Missing Response Options (*N* = 12)	Issues with Sequence of Response Options (*N* = 12)	Recall Period Used (*N* = 12)	Recall period when not used: Today possible? ^b^	Item Relevance (*N* = 12)
Sect. 1- Pain intensity	11/12 aligned 1/12 related	12/12 easy to understand	8/10 aligned interpretation 2/10 reported 2 options as similar	11/12 none 1/12 ability to work	12/12 none	10/12 Today 1/12 Past two years 1/12 Overall/in general	2/2 Yes	11/12 Relevant 1/12 Provide Night time impact
Sect. 2- Personal care (washing, dressing, etc.)	12/12 aligned	11/12 easy to understand 1/12 found options were lengthy	9/10 aligned interpretations 1/10 misinterpreted	11/12 none 1/12 mild pain intensity looking after myself	11/12 none 1/12 Move option #2 after #3	6/12 Today 4/12 Overall/ in general 1/12 Past week 1/12: Past 10 years	6/6 Yes	11/12 Relevant 1/12 “Flare” related
Sect. 3- Lifting	12/12 aligned	12/12 easy to understand	7/10 aligned interpretations 3/10: confused or found 2 options as similar	10/12 none 1/12 Clarification due to back pain or other 1/12 Definition for heavy, medium, and light weights	12/12 none	5/12: Today 5/12: Overall/ in general 2/12: Past week	5/7 Yes 2/7 Have not lifted anything yet that day	11/12 Relevant 1/12 Avoids activity
Sect. 4- Walking	12/12 aligned	11/12 easy to understand 1/12 Preferred spatial references or duration of walking	10/10 aligned interpretations	10/12 none 1/12: Half a mile 1/12: Being in bed most of the time, but could walk very short distances	12/12 none	4/12: Today 4/12: Overall/ in general 2/12: Past week 1/12: Past few months 1/12: More than 1 year ago	7/8 Yes 1/8 Could not respond thinking about today as day just started	11/12 Relevant 1/12 not impacted
Sect. 5- Sitting	12/12 aligned	12/12 easy to understand	10/10 aligned interpretations	11/12 none 1/12 Use of assistive pillows	12/12 none	7/12: Today 2/12: Overall/ in general 1/12: Past week 1/12: Past month 1/12: >past year	5/5 Yes	12/12 Relevant
Sect. 6- Standing	12/12 aligned	12/12 easy to understand	10/10 aligned interpretations	12/12 none	12/12 none	6/12: Today 2/12: Past week 2/12: Overall/ in general 1/12: Last few months 1/12: Past 2 years	5/6 Yes 1/6 not asked	12/12 Relevant
Sect. 7- Sleeping	12/12 aligned	12/12 easy to understand	8/10 aligned interpretations 2/10 misinterpreted 1 option	12/12 none	12/12 none	7/12: Today/last night 4/12: Overall/ in general 1/12: Past 2 years	5/5 Yes	11/12 Relevant 1/12: Not impacted
Sect. 8- Sex life	11/12 aligned 1 broad interpretation	12/12 easy to understand	5/10 aligned interpretations 5/10 found similarities in 2–3 options, or found 1 option difficult to interpret	11/12 none 1/12 Short-term relief immediately after sexual activity	12/12 none	2/12: Today 4/12 Overall/in general 2/12: In the past week 2/12: In the past few months 2/12: A year ago or longer	9/10 Yes 1/10 could not respond based on today	9/12 Relevant 3/12 Not very relevant or applicable
Sect. 9- Social life	11/12 aligned 1 paraphrased a response option	12/12 easy to understand	7/10 aligned interpretations 3/10 found similarities in 2 options or did not indicate difference in 1 option	12/12 none	12/12 none	2/12: Today 3/12: Past week 4/12 Overall/ in general 1/12 Past month 2/12 A year ago or longer	9/10 Yes 1/10 could not respond based on today because they did not leave home yet	12/12 Relevant
Sect. 10- Traveling	12/12 aligned	12/12 easy to understand	8/10 aligned interpretations 2/10 found similarities in 2 options, or did not see relevance in 2 options	12/12 none	12/12 none	2/12: Today 4/12: Overall/ in general 4/12: Past week 2/12: A year ago or longer	5/10 Yes 4/10 could not respond because they did not leave home yet 1/10 not asked	12/12 Relevant

^a^Two participants are not included in the total N as one was not asked these questions and another participant was unable to paraphrase the options

^b^The total N corresponds to the number of participants who did not use the correct recall period (i.e., “Today”) when answering a section

#### Section 1: Pain Intensity

When discussing the Pain Intensity section, the majority of participants did not have any issues or difficulties. All participants reported the response options easy to understand (*n* = 12, 100%) and most (11/12, 92%) provided responses supportive of a correct interpretation of the question, for example:

How intense the pain is right now.

The majority of participants were aligned in their interpretations of the response options (8/10, 80%) and reported the response options to be comprehensive (11/12, 92%). All participants (12/12, 100%) reported no issues with the sequence of the response options. Nearly all participants (11/12, 92%) thought the pain intensity item was relevant to their experience, while one suggested they experience back pain mostly overnight, thus implying the “today” element did not capture their night-time experience.

When discussing the recall period, the majority of participants (10/12, 83%) answered using the correct ‘today’ recall. Two participants (2/12, 17%) reported answering based on the past 2 years and their overall daily experience, respectively.

#### Section 2: Personal Care

When discussing the Personal Care section, the majority of participants did not have any issues or difficulties, other than the use of the correct recall period. All participants demonstrated they were aligned in understanding the question interpretation (12/12, 100%), for instance:

Everyday things, such as taking a shower, dressing myself, doing my hair.

The majority of participants reported the response options easy to understand (11/12, 92%), were aligned in their interpretations of the response options (9/10, 90%), stated there were no missing options (11/12, 92%), had no issues with the sequence of options (11/12, 92%). Most (11/12, 92%) indicated that the personal care item was relevant to their experience:

It is very relevant, I always have back pain when I take a shower. It does not stop me from taking a shower but it is painful.

Half of the participants (*n* = 6/12, 50%) used the correct recall, while the remaining six participants answered thinking of the past week (*n* = 1, 8%), the past ten years (*n* = 1, 8%) or their overall experience (*n* = 4/12, 33%).

#### Section 3: Lifting

Overall, the majority of participants did not have any issues or difficulties with this section, with the exception of answering using the correct recall. All participants (12/12, 100%) correctly interpreted the question (reflecting movement, positioning, and/or extent of weight), for instance:

How much you can lift with or without pain.

All participants (12/12, 100%) reported the response options easy to understand and had no issues with the sequence of options (12/12, 100%). Most stated that there were no missing options (10/12, 83%) and were aligned in their interpretations of the response options (7/10, 70%). The majority (11/12, 92%) reported the lifting item relevant to their experience.

Less than half of the sample were thinking of ‘today’, as instructed (5/12, 42%). The remaining seven participants used a variety of recall periods, including the past week (*n* = 2, 17%) or their overall experience (*n* = 5, 42%). Three participants indicated that it would be easier to answer this lifting section thinking of the past week.

#### Section 4: Walking

Overall, for the Walking section, the majority of participants did not have any issues or difficulties, with the exception of using the correct recall period. All participants provided interpretations supportive of a correct interpretation (reflect distance or impact on walking), for example:

How the pain affects your walking.

The majority of participants reported the response options easy to understand (11/12, 92%), were aligned in their interpretations of the response options (10/10, 100%) and indicated that there were no missing options (10/12, 83%). All participants (12/12, 100%) supported the sequence of response options. The majority (11/12, 92%) thought the walking item was relevant to their experience.

One-third of participants (4/12, 33%) used the correct recall. Eight participants used various recall periods, including the past week (*n* = 2, 17%), the last few months (*n* = 1, 8%), more than a year ago (*n* = 1, 8%), or their overall experience (*n* = 4, 33%).

#### Section 5: Sitting

When discussing the Sitting section, the majority of participants did not have any issues or difficulties. All 12 participants reported the response options easy to understand and provided interpretations supportive of a correct interpretation of the item (reflecting sitting posture and/or duration):

How long you can sit properly without having to get up.

All or the majority of participants were aligned in their interpretations of the response options (10/10, 100%), stated there were no missing options (11/12, 92%), had no issues with the sequence of options (12/12, 100%) and reported sitting as relevant to their experience (12/12, 100%).

Just over half (7/12, 58%) of participants answered the item using the correct recall period. Five participants used a variety of recall periods, including the past week (*n* = 1, 8%), the past month (*n* = 1, 8%), longer than the past year (*n* = 1, 8%) or their overall experience (*n* = 2, 17%).

#### Section 6: Standing

Overall, for the Standing section, the majority of participants did not have any issues or difficulties, with the exception of the recall period. All participants (12/12, 100%) reported the response options easy to understand and provided interpretations supportive of a correct interpretation (reflecting standing posture and/or duration), for instance:

How well can you stand without pain.

All participants were aligned in their interpretations of the response options (10/10, 100%), reported no missing options (12/12, 100%), had no issues with the sequence of options (12/12, 100%), and reported standing as relevant to their experience (12/12, 100%).

In terms of the recall period used, half of the participants (6/12, 50%) reported considering today as intended when asked the time period they had in mind, while the remaining participants answered using the past week (*n* = 2, 17%), the last few months (*n* = 1, 8%), the past 2 years (*n* = 1, 8%), and their overall experience (*n* = 2, 17%).

#### Section 7: Sleeping

Overall, for the Sleeping section, the majority of participants did not have any issues or difficulties. All participants (12/12, 100%) reported the response options easy to understand and provided interpretations supportive of a correct interpretation (reflecting sleep interruption and/or duration), for example:

How your pain is affecting your sleep.

All or the majority had no issues with the sequence of options (12/12, 100%) and reported sleeping as relevant to their experience (11/12, 92%). Most were aligned in their interpretations of the response options (8/10, 80%).

Just over half (7/12, 58%) participants reported considering today/last night as intended, while the remaining 5 participants reported answering based on their overall experience (*n* = 4, 33%) or the past two years (*n* = 1, 8%).

#### Section 8: Sex Life

When discussing the Sex Life section, participants generally did not have any issues or difficulties, other than with their interpretations with some response options and the recall period. All participants (12/12, 100%) reported that they thought the response options were easy to understand and almost all of them (11/12, 92%) provided interpretations supportive of a correct interpretation (reflecting sexual activity and interference), for instance:

My sex life with my husband and pain interfering with that.

All or the majority indicated that there were no missing options (11/12, 92%) and had no issues with the sequence of options (12/12, 100%). Only half were aligned in their interpretations of the response options (5/10, 50%) with most issues related to participants’ perceived similarities between certain response options. Four participants reflected issues with the similarity of the response options, between the fourth and fifth options (“My sex life is severely restricted by pain” and “My sex life is nearly non-existent because of pain”), while two additionally mentioned the sixth option (“Pain prevents me from having any sex life at all”). Moreover, two participants had difficulties interpreting as they could not understand how a sex life can be “nearly normal” and “very painful” at the same time, as per description of the third response option.

Few (2/12, 17%) participants reported answering using the ‘today’ recall for this item. The majority answered using various recall periods, including the past week (*n* = 2/12, 17%), the past few months (*n* = 2/12, 17%), a year ago or longer (*n* = 2/12, 17%), and their overall experience (*n* = 4/12, 33%). Three quarters of participants (9/12, 75%) thought the sex life item relevant to their experience, while the remaining three participants (25%) did not find this item very relevant or applicable to their life.

#### Section 9: Social Life

When discussing the Social Life section, the majority of participants did not have any issues or difficulties, other than with the recall period. All participants (12/12, 100%) reported that the response options were easy to understand and most (11/12, 92%) provided interpretations supportive of a correct interpretation (reflecting degree or type of social activity), for example:

How it impacts my social life. I used to have one […] I don’t anymore.

All participants stated there were no missing options (12/12, 100%), had no issues with the sequence of options (12/12, 100%), and reported social life as relevant to their experience (12/12, 100%). Most were aligned in their interpretations of the response options (7/10, 70%).

Only two participants reported considering today as intended. The majority (10/12, 83%) answered this item recalling the past week (*n* = 3/12, 25%), the past month (*n* = 1/12, 8%), a year ago or longer (*n* = 2/12, 17%), or their overall experience (*n* = 4/12, 33%).

#### Section 10: Traveling

Overall, for the Traveling section, the majority of participants did not have any issues or difficulties, other than with the recall period. All participants (12/12, 100%) reported that they thought the response options were easy to understand and provided interpretations supportive of a correct interpretation (reflecting duration or distance and/or travel activities), for instance:

Being able to travel with zero pain to high level of pain.

All participants (12/12, 100%) stated there were no missing options, had no issues with the sequence of options, and reported travelling as relevant to their experience. Most were aligned in their interpretations of the response options (8/10, 80%).

Few (2/12, 17%) participants reported answering the item based on ‘today’ as intended. The majority (10/12, 83%) answered using alternative recall periods, including the past week (*n* = 4/12, 33%), a year ago or longer (*n* = 2/12 17%), or their overall experience (*n* = 4/12, 33%).

#### Preferred recall period

Due to issues identified during the first three interviews in using the correct recall period (i.e., today) when answering the ODI, the remaining nine participants were asked a question on the preferred recall period overall at the end of the interview. Most indicated that it would be easier to think of the past week when answering this questionnaire (7/9, 78%), while the remaining two preferred a recall period of ‘today’.

#### Concept saturation

During the interviews, participants were asked if there were important concepts they would include in the ODI. Suggestions included adding a section on bending or twisting, however, upon probing, participants suggested these concepts were covered by existing sections within the ODI, such as the personal care section. Three participants suggested to add some questions to obtain further background information of the participant. The proposed questions covered pain relief aspects, other comorbid conditions, and the use of pain medication; however, these concepts are not related to the intent of the ODI (i.e., measuring pain-related disability).

After the completion of the 10th interview, additional interviews were needed to establish concept saturation. The 12th interview discussed unique concepts that reflected variation in the interviewee’s individual life context. New concepts included an upcoming breast reduction surgery to make tasks easier (this can be related to concept of comorbidity); the concept of bending being different from lifting because it brings some pain relief; and a feeling of relief following sexual activity (activities of relief are not intended to be part of ODI). It is unlikely that additional interviews would have identified new concepts or additional results that would change the overall interpretation of the ODI.

## Discussion

The ODI was originally developed to assess pain intensity and pain-related disability in patients with diseases of the lumbar spine. The present study aimed to assess the content validity of the electronic ODI v2.1.b (US English) specifically among patients who reported they had been diagnosed with DDD to ensure that the instrument is suitable for measuring pain intensity and pain-related disability in a future clinical trial setting involving patients with DDD.

A recent systematic review stated that the ODI represents “the gold standard metric used in lumbar degenerative disease” [50] and another confirmed the suitability for use of the ODI as they concluded the ODI was strongly recommended for use in measuring physical functioning domain in Low Back Pain [40]. The evidence from this study supports the content validity of the ODI for use as an outcome to define endpoints in clinical trials. Our results showed nearly all participants (11/12, 92%) indicated that the ODI was thorough and comprehensive in evaluating their experiences living with DDD. Participants correctly interpreted all section meanings and reported the ODI easy to understand. The concepts covered by the ODI were relevant to ≥ 11/12 participants (≥ 92%), with the exception of the sex life (Sect. 8), which was relevant to 9/12 participants (75%).

Suggestions included adding a section on bending or twisting; however, these concepts may be covered with existing sections within the ODI. Three participants suggested adding questions to obtain further background information of the participant, including about what factors alleviate or worsen their back pain, other comorbid conditions affecting their back pain, and their dependency on pain medication due to back pain, information that can be captured at baseline rather than as outcomes. Concept saturation was reached on the 12th interview.

The correct recall period was used variably across the sections ranging from 2/12 − 10/12 (17%- 83%) participants, with participants noting challenges in nine of the 10 conceptual areas of ODI. The specified daily recall was least used in the sex life, social life and travelling sections. The majority of participants (7/9, 78%) preferred answering the ODI using a one-week recall.

For patients with DDD, pain intensity and pain-related disability are important outcomes to consider when evaluating the effectiveness of interventions in a clinical trial setting. Since these concepts are known only to patients, the measurement of these outcomes is often most accurately and reliably assessed by patients’ self-reports.

There has been substantial evidence that pain intensity and pain-related disability as defined by personal care, lifting, walking, sitting, standing, sleeping, sex life, social and travel are concepts of interest to patients with CLBP [12, 18, 37, 39]. Furthermore, findings indicate that these are relevant domains that should be evaluated when assessing a therapeutic response in patients with CLBP [12, 18, 39].

While there are several PROMs specific to CLBP [13, 41, 43], many were only utilized in one or two studies and do not meet the standards established by the current scientific literature and in FDA Guidance [51] due to insufficient data to support content validity, construct validity, and ability to detect change or defined MCID [13, 41, 43]. The ODI has been recommended by various groups (e.g., WHO, ICHOM, IMMPACT) as an important PROM in patients with CLBP due to its frequency of use and evidence of validity [13, 36–39, 41, 43].

The content validity of the ODI is well-established. Waddell and colleagues conducted interviews with 182 patients with CLBP referred to a specialty back pain clinic to establish concepts of interest for this patient population [18]. Eight of the nine domains identified by Waddell and colleagues map to concepts in the ODI, including sitting, lifting, traveling, standing, walking, sleep, social life, and sex life, with the only exception being “help with footwear” which ODI captures with in the Personal Care section. Furthermore, Turk and colleagues further confirmed the content validity of the ODI in a study aimed at identifying important concepts of interest in patients with CLBP using patient focus groups and a patient survey [12]. The concepts of interest identified in Turk’s study mapped to the content of the ODI, specifically physical activities, fatigue, participating in social and family activities, household activities, and sex life [12]. However, a recent systematic review highlighted the urgent need for a qualitative assessment of the ODI’s relevance, comprehensiveness, and comprehensibility for measuring physical functioning in this patient population [44].

This cognitive interview study has addressed this need and added to the understanding of the content validity of the ODI, specifically the electronic ODI v2.1.b (US English) in patients with DDD. Overall, the results of this study show the relevance and ease of understanding of the ODI instructions, items, and response options for patients with DDD. The ODI was reported to be comprehensive, with saturation of concepts being achieved by the 12th interview. Where suggestions were indicated, participants indicated these as contained in existing sections (twisting, bending) or were suggestions that would be best addressed by a separate measure (dependency on medication and comorbidities).

The primary issue identified during the interviews was with the recall period used by participants when answering the items with participants favoring a longer recall period in a chronic illness. The instructions in the screenshots of the ODI reviewed in the interview included the instruction to “select the answer that most closely describes you today”; however, participants changed their recall based on the question being asked. Recall periods used by participants when responding to the items included “today”, the “past week”, over the past few months, and just their experiences with DDD “in general.” Verbal reminders by an ODI administrator may help to ensure participants focus on ‘today’ as required the recall period. However, in a trial setting, this may not be practical for implementation as it does not appear that the ODI can be administered by an external interviewer [52]. Furthermore, certain activities such as sexual activity, social functions, and traveling, may not be applicable on a daily basis in a chronic illness where events may not accrue or change daily. As such, it may be more meaningful to modify and extend the recall period for use in this population. This change would also be in line with results on recall period preferences, as a systematic review of qualitative studies showed patients had minimal concerns over the accuracy of a one-week recall period and highlighted patients’ difficulty to adhere to a recall period of “today” [53].

A systematic review of PROMs in general by Peasgood and colleagues [53] found a seven-day recall period would lead to more severe pain and lower health related quality of life reported by respondents. However, an increased recall period to one week is supported by the results from this study. First, most participants asked (7/9, 78%) confirmed their preference for a one-week recall period. The instructed recall period of ‘today’ was identified in early interviews as an issue and therefore, the study team was able to explore alternatives in subsequent interviews and gain a strong indication that a 7-day recall period would be more appropriate for this patient group. Second, some participants answered items using the one-week recall period. Third, participants indicated that they were not able to answer certain sections using the “today recall” as they did not complete the task yet due to the timing of the interview (e.g., traveling or sexual activity), or did not consider the activities as being encapsulated in ‘today’ (e.g., night time). Therefore, while the concepts in the ODI are relevant and appropriate for patients with DDD, modifying the recall to a seven-day recall period is recommended for this population.

While this study provided novel insights into the content validity of the ODI, it does have some limitations. The study sample were required to confirm they had been diagnosed with DDD, and that they had lower back pain for 6 months or longer that impacted their everyday abilities as part of the consent process, but were not required to provide additional verification materials such as a clinician letter. Another limitation is the overall sample diversity, as there was a majority of female (11/12, 92%) and White/Caucasian (10/12, 83%) participants, with an age limited to 18–55 years per eligibility criteria. While it is advised to interpret these results cautiously in light of this, being White/Caucasian and female have been identified as important risk factors in this condition, especially in a younger population [54].

It is also important to note that some eligibility criteria were based on the planned population of an upcoming Phase III trial, namely those with lower back pain for at least 6 months with related disability. This study additionally focused on US-English speaking participants who were able to be at and use a computer. Further assessment of the suitability of the ODI is recommended for any studies with other populations of interest, such as an older age, more diverse group or non-English speaking individuals.

## Conclusion

Based on current scientific methodology for assessing an existing PROM this study demonstrated that (1) there is conceptual alignment between the ODI and the desired trial population; (2) we obtained direct patient input from the target population for the trial; (3) we provided evidence that the ODI contains item content that is most relevant and important to patients with DDD and (4) we provide the evidence to support a change in the recall period of the ODI.

This study confirms the ODI to be easy to understand and relevant in patients with DDD. The ODI is comprehensive with the 10 sections covering concepts important in this population. The majority had no issues with most of the elements explored, in respect to the intent of the questionnaire and the sections, had no difficulty in understanding the ODI instructions and section response options, reported no issues with the sequence of the response options, and reported no missing response options. However, there were challenges with using the ‘today’ recall consistently throughout the questionnaire. Based on patient feedback, an ODI version with a seven-day recall period would be more appropriate in this population in order to directly evaluate patient outcomes and treatment benefits in clinical trials.

## Data Availability

De-identified data may be available from the corresponding author upon request.

## Abbreviations

CLBP: Chronic Low Back Pain
DDD: Degenerative Disc Disease
ICHOM: International Consortium for Health Outcomes Measurement
IMMPACT: Initiative on Methods, Measurement and Pain Assessment in Clinical Trials
IRB: Independent Review Board
LBP: Lower Back Pain
MCID: Minimal Clinical Important Difference
mODI: Modified Oswestry Disability Index
MRI: Magnetic Resonance Imaging
NRS: Numeric Rating Scale
ODI: Oswestry Disability Index
PROMs: Patient-Reported Outcome Measures
SD: Standard Deviation
VAS: Visual Analog Scale
WHO: World Health Organization
